# Migraine and body mass index categories: a systematic review and meta-analysis of observational studies

**DOI:** 10.1186/s10194-015-0510-z

**Published:** 2015-03-28

**Authors:** Raffaele Ornello, Patrizia Ripa, Francesca Pistoia, Diana Degan, Cindy Tiseo, Antonio Carolei, Simona Sacco

**Affiliations:** Department of Applied Clinical Sciences and Biotechnology, Institute of Neurology, University of L’Aquila, 67100 L’Aquila, Italy

**Keywords:** Migraine, Obesity, Body mass index

## Abstract

**Background:**

Several studies have assessed the associations between migraine and underweight, pre-obesity or obesity, with conflicting results. To assess the consistency of the data on the topic, we performed a systematic review and meta-analysis of the available observational studies.

**Methods:**

Multiple electronic databases were systematically searched up to October 2014 for studies assessing the association between migraine and body mass index categories (underweight, pre-obesity, or obesity).

**Results:**

Out of 2,022 records, we included 15 studies. When considering the 11 studies following the World Health Organization BMI cutoffs, we found an increased risk of having migraine in underweight subjects (pooled adjusted effect estimate [PAEE] 1.21; 95% CI, 1.07-1.37; P = 0.002) and in obese women (PAEE 1.44; 95% CI, 1.05-1.97; P = 0.023) as compared with normal weight subjects; additionally, pre-obese subjects had an increased risk of having chronic migraine (PAEE 1.39; 95% CI, 1.13-1.71; P = 0.002). When considering all the 15 studies, we additionally found an increased risk of having migraine in obese as compared with normal weight subjects (PAEE 1.14; 95% CI, 1.02-1.27; P = 0.017); additionally, obese subjects had an increased risk of having chronic migraine (PAEE 1.75; 95% CI, 1.33-2.29; P < 0.001). The pooled analysis did not indicate an increased risk of having migraine in pre-obese subjects.

**Conclusions:**

The meta-analysis of the available observational studies suggested an association between migraine and obesity likely mediated by gender and migraine frequency. Further studies taking into account gender, migraine type, frequency, activity, and duration could provide more robust evidence.

**Electronic supplementary material:**

The online version of this article (doi:10.1186/s10194-015-0510-z) contains supplementary material, which is available to authorized users.

## Background

Migraine has a prevalence of about 11% in the general population and is the seventh most disabling disease worldwide [1,2]. Obesity has had an increasing prevalence in recent decades and is associated with established health risks and increased mortality [3]. While a large number of epidemiological studies have investigated the association between migraine and obesity [4,5], their conflicting results have hindered any definite conclusion about a possible increased risk of having migraine for obese subjects or a possible increased risk of being obese in the presence of migraine. Obesity has, however, been found to be associated with high headache frequency [6] and to be involved in the transformation of migraine from episodic to chronic [7]. The relationship between migraine and obesity is complex and still unclear, as the two conditions share some pathogenic determinants and may influence one another [6].

In order to clarify and quantitatively reappraise the epidemiological evidence of any link between migraine and body mass index (BMI) categories, we performed a meta-analysis of the available observational studies.

## Methods

The present meta-analysis was performed according to the PRISMA guidelines [8].

### Eligibility criteria

We included in the analysis published studies meeting all the following predetermined criteria: 1) any migraine or chronic migraine as outcome variables, and underweight, pre-obesity, or obesity as compared with normal weight range as exposure variables, or *vice versa*; 2) an observational design; 3) a clearly reported, unequivocal definition of exposure and outcome variables (migraine, chronic migraine, underweight, overweight, pre-obesity, obesity); 4) an adjusted statistical model or a matching procedure to control for potential confounders; 5) the report of effect estimates with 95% confidence intervals (CIs); and 6) English language. Therefore, we excluded studies: 1) investigating non-migraine headaches; 2) considering BMI values as a matching factor in case-control studies and not as a variable in their statistical model; 3) comparing mean BMI values without providing effect estimates for BMI categories; 4) with an interventional design (such as randomized controlled trials), case reports, and case series; 5) including only subjects with migraine, only overweight or obese subjects, or only subjects with metabolic syndrome; 6) reporting only abstracts and unpublished material; or 7) not published in English. We also excluded from the analyses studies performed in children and adolescents, because of the potential bias due to the lower prevalence and more balanced gender distribution of migraine in those age groups as compared with adults [1].

### Information sources and search strategy

Two investigators (PR and RO) independently searched papers indexed in PubMed, Science Citation Index, and Scopus from the beginning of indexing to October 31, 2014 and containing the terms “migraine”, “headache”, or “headache disorder” combined with the terms “obesity”, “overweight”, “body fat”, “body mass index”, or “waist circumference”. Filters for English language were applied. In PubMed, the terms were searched as both text words and Medical Subject Heading (MeSH) terms. A manual search among references of selected articles and reviews was also performed (PR and RO).

### Study selection and data collection

In the review process we followed a two-step procedure. In the first step, two investigators (CT and DD) independently reviewed titles and abstracts of the potentially eligible papers. In the second step, the same investigators (CT and DD) independently reviewed the full-text version of the selected articles and performed the manual search of the references. In both steps, all disagreements were resolved by consensus among all the researchers.

A standardized form was used for data extraction, including the following items: first author, year of the study, country where the study was performed, study design and cohort, period of subject enrollment, age range, gender distribution, exposures, outcomes, number of migraineurs and of underweight, pre-obese, and obese subjects according to BMI categories, type of effect estimate, and confounders assessed.

### Assessment of the risk of bias in individual studies

To ascertain the quality of eligible studies, we applied the criteria of the Newcastle-Ottawa Scale for observational studies [9] with modified criteria for cross-sectional studies [10]. We considered the risk of bias as low, medium, or high if the included studies did not fulfill one, two, or three of those criteria, respectively.

### Statistical analysis

We used odds ratios (ORs) and hazard ratios (HRs) to estimate effect size. We chose to pool the adjusted effect measures rather than the crude ones because of the role of confounding factors on the validity of observational studies. When different adjusted models were available, we chose the model including the largest number of factors. We performed an overall analysis of the association between the selected exposure and outcome variables. Where possible, we also performed subgroup analyses for gender. We assessed the association between migraine and BMI categories, performing separate analyses for the studies regarding migraine as the outcome and BMI categories as the exposure and for the studies regarding BMI categories as the outcome and migraine as the exposure. To obtain the pooled adjusted effect estimate (PAEE), the natural logarithm of each single estimate was weighted by the inverse of its variance. We ran a random effects model [11] rather than a fixed effects one because of the high likelihood of between-study variance. To reduce methodological heterogeneity we performed our primary analyses including only the studies which defined BMI categories according to the World Health Organization (WHO) criteria for Western populations (underweight, <18.50 kg/m^2^; normal range, 18.50 - 24.99 kg/m^2^; overweight, ≥25.00 kg/m^2^; pre-obesity, 25.00 - 29.99 kg/m^2^; obesity, ≥30.00 kg/m^2^) [12]. Together with those primary analyses performed with narrow inclusion criteria, we performed additional analyses with broad inclusion criteria including studies with BMI cutoffs other than those of the WHO. When performing analyses including the studies fulfilling broad criteria, we considered the mid-points of BMI categories as moderators in the statistical models in order to correct for the different cutoffs. We also performed a linear analysis by transforming category-specific risk estimates into estimates of the effect size associated with every 5 kg/m^2^ increase in BMI in studies fulfilling broad inclusion criteria by use of the method of generalized least-squares for trend estimation [13]; the value assigned to each BMI category was the mid-point for closed categories, 18 for underweight, and 35 for obesity, with the last two values taken from a previous paper [14] because of the impossibility of calculating means or medians for the study populations.

In accordance with the Cochrane Collaboration Guidelines for systematic reviews [15], we assessed the clinical, methodological, and statistical heterogeneity of the included studies. Clinical heterogeneity was assessed by evaluating differences in the study populations, exposures, and outcomes. Methodological heterogeneity was assessed by comparing the differences among the adjusted models. Statistical heterogeneity was assessed using the I^2^ statistic [15]. We performed a sensitivity analysis to quantify the effect of each of the included studies (through a “leave-one-out” method) and of low quality studies (not fulfilling three quality criteria) on the overall results. Analyses were carried out with *R* software [16] using the *meta* [17] and *metafor* [18] packages.

## Results

The electronic database search retrieved 2,022 records. Additional file 1 displays the review process. After reviewing titles and abstracts we identified 41 eligible papers for full-text review. The manual search retrieved 5 further papers. Thirty-one studies [6,14,19-47] were excluded: 6 of them [14,19-23] did not define or assess the variables of interest; 21 studies [6,24-43] did not report extractable data on the variables of interest; 1 study [44] included only adolescents; 1 [45] did not define obesity by means of BMI categories; 1 [46] reported data only on migraine subtypes; 1 [47] had a design which rendered its data incomparable with the other studies. Four studies [48-51] fulfilled broad but not narrow inclusion criteria: 3 of them [48-50] had BMI categories different from the WHO standards, while 1 [51] took BMI categories from the WHO standards for Asian people. We finally included 11 studies in the pooled analyses according to narrow criteria [52-62] and 15 studies according to broad criteria [48-62].

### Study characteristics

Table 1 displays the characteristics of the studies fulfilling narrow inclusion criteria, while Table 2 describes those fulfilling only broad inclusion criteria. One of the studies [62] had a cohort design, investigated an exclusively female population, and had BMI category as the outcome. Among the 14 cross-sectional studies, thirteen [48-54,56-61] had migraine as the outcome and BMI categories as the exposure; three of those studies [49,57,61] included women only, eight [48,50,51,53,54,56,59,60] reported the overall results for both men and women, and three [52,56,58] reported their results according to gender. One of the cross-sectional studies [55] had BMI category as the outcome and migraine as the exposure and did not report separate results according to gender.

**Table 1:** **Main characteristics of the studies fulfilling narrow inclusion criteria (definition of body mass index categories according to the World Health Organization criteria)**

**Study**	**Country**	**Design (population)**	**Period of inclusion**	**Age range (years)**	**Women (%)**	**Exposure(s)**	**Outcome(s) of interest**	**Migraineurs (n, %)**	**Underweight (n, %)**	**Pre-obese (n, %)**	**Obese (n, %)**	**Type of effect estimate**	**Confounders**
Bigal, 2006 [52]	USA	Cross-sectional	1997-2000	≥18	65.0	Underweight, pre-obesity, obesity	Any migraine	3,791 (12.5)	941 (3.1)	9,258 (30.6)	3,133 (10.4)	OR	Marital status, income, use of daily medications, depression
Bigal and Lipton, 2006 [53]	USA	Cross-sectional	1997-2000	≥18	61.8	Underweight, pre-obesity, obesity	Transformed migraine	(1.3)	941 (3.1)	9,258 (30.6)	3,133 (10.4)	OR	Use of acute or preventive medication, age, race, socioeconomic status, depression
Gilmore, 1999 [54]	Canada	Cross-sectional* (NPHS)	1996-1997	20-64	49.4	Underweight, pre-obesity, obesity	Any migraine	NR	NR	(34)	(12)	OR	Age, sex
Jiménez-Sánchez, 2013 [55]	Spain	Cross-sectional (SNHS-NHSRP)	2006	≥16	50.9 (Spanish), 53.1 (Romany)	Any migraine	Obesity	(12.5, Spanish; 29.4, Romany)	-	-	(14.6, Spanish; 21.2, Romany)	OR	Gender, age, education level, occupational status, self-related health, smoking habit, alcohol consumption, sleep habits, physical exercise, hypertension, asthma, heart disease, osteoarthritis, allergy, diabetes, stomach ulcer, hypercholesterolemia,depression, osteoporosis, prostate problems, menopausal symptoms
Le, 2011 [56]	Denmark	Cross-sectional (DTR)	2002	20-71	69.6	Underweight, pre-obesity, obesity	Any migraine	8,044 (25.2)	230 (3.0) [migraine]; 477 (2.1) [healthy]	2,072 (27.2) [migraine]; 6,973 (30.8) [healthy]	682 (9.0) [migraine]; 1,743 (7.7) [healthy]	OR	Age, education, employment status, physical work load, recreational physical activity, smoking status, alcohol consumption, marital status
Mattsson, 2007 [57]	Sweden	Cross-sectional	1997-1998	40-74	100.0	Obesity	Active migraine	130 (19.0)	-	-	(19.3)	OR	Age, education
Peterlin, 2010 [58]	USA	Cross-sectional* (NHANES)	1999-2004	≥20	51.2	Obesity	Any migraine	4,664 (21.4)	-	-	6,504 (29.9)	OR	Age, income, education, race, smoke, alcohol use, diabetes
Peterlin, 2013 [59]	USA	Cross-sectional (NCS-R)	2001-2003	≥18	50.3	Underweight, pre-obesity, obesity	Episodic migraine	188 (4.9)	129 (3.3)	1,306 (33.8)	1,004 (26.0)	OR	Age, race, sex, smoking, poverty index, depression, diabetes
Santos, 2014 [60]	Brazil	Cross-sectional* (ELSA-Brazil)	2008-2010	35-74	54.0	Pre-obesity, obesity	Non-daily migraine, daily migraine	4,047 (27.8) [non-daily]; 228 (1.6) [daily]	-	5,915 (40.6)	3,308 (22.7)	OR	Age, sex, race, education level, monthly income, smoking status, diabetes, hypertension, medication use
Vo, 2011 [61]	USA	Cross-sectional* (Omega Study)	1996-2008	≥18	100.0	Underweight, pre-obesity, obesity	Any migraine	670 (17.9)	160 (4.3)	612 (16.4)	338 (9.1)	OR	Age, race/ethnicity, educational attainment, marital status, history of hypertension, history of diabetes mellitus, smoking, exercise status
Winter, 2012 [62]	USA	Cohort (WHS)	1992-1995	≥45	100.0	Any migraine, MO, MA	Pre-obesity, obesity	3,483 (18.2)	-	7,916 (41.3)	730 (3.8)	HR	Age, race, randomized treatment assignments, baseline BMI, total calorie intake, alcohol consumption, exercise, smoking status, postmenopausal hormone use, postmenopausal status, history of cholesterol >240 mg/dl, history of hypertension, history of depression

*baseline cross-sectional analysis of a cohort study.

BMI, body mass index; DTR, Danish Twin registry; ELSA, Longitudinal Study of Adult Health; HR, hazard ratio; NCS-R, National Comorbidity Survey Replication; NHANES, National Health And Nutrition Examination Survey; NHSRP, National Health Survey in the Romany Population; NPHS, National Population Health Survey; NR, not reported; OR, odds ratio; SNHS, Spanish National Health Survey; WHS, Women’s Health Study.

**Table 2:** **Main characteristics of the studies fulfilling only broad inclusion criteria (no definition of body mass index categories according to the World Health Organization criteria)**

**Study**	**Country**	**Design (population)**	**Period of inclusion**	**Age range (years)**	**Women (%)**	**Exposure(s)**	**Outcome(s) of interest**	**BMI categories**	**Migraineurs (n, %)**	**Underweight (n, %)**	**Pre-obese (n, %)**	**Obese (n, %)**	**Type of effect estimate**	**Confounders**
Queiroz, 2009 [48]	Brazil	Cross-sectional	2006-2007	18-79	60.0	Pre-obesity, obesity	Any migraine	<25; 25.0-29.9; ≥30.0	627 (15.2)	-	1,147 (29.8)	484 (12.6)	PR	Gender, age, education level, marital status, household income, job status, physical activity
Winter, 2009 [49]	USA	Cross-sectional* (WHS)	1992-1995	≥45	100.0	Obesity	Active migraine	<23.0; 23.0-24.9; 25.0-26.9; 27.0-29.9; 30.0-34.9; ≥35.0	9,195 (14.5)	-	-	1,206 (13.1)	OR	Age, smoking, exercise, alcohol consumption, history of hypertension, postmenopausal status, postmenopausal hormone use, history of elevated cholesterol
Winter, 2011 [50]	Germany	Cross-sectional (DHS, KORA, SHIP)	NR (DHS); 1994-1995 (KORA); 1997-2001 (SHIP)	25-75 (DHS); 35-75 (KORA); 25-88 (SHIP)	52.9 (DHS); 52.2 (KORA); 51.9 (SHIP)	Pre-obesity, obesity	Complete migraine	<25; 25.0-29.9; ≥30.0	91 (8.5) [DHS]; 222 (7.9) [KORA]; 114 (4.3) [SHIP]	-	NR	NR	OR	Age, gender
Yu, 2012 [51]	China	Cross-sectional	2009	18-65	49.2	Underweight, pre-obesity†, obesity†	Any migraine	<18.5; 18.5-22.9; 23.0-24.9; 25.0-29.9; ≥30.0	467 (9.3)	262 (5.2)	1,044 (20.8)	190 (3.8)	OR	Age, gender, habitation, marital status, occupation, education level

*baseline cross-sectional analysis of a cohort study.

†the authors of that paper used the terms “obesity” and “morbid obesity” to define BMI 25.0-29.9 kg/m^2^ and BMI≥30 kg/m^2^, respectively; we used BMI-based definitions instead of the given ones.

BMI, body mass index; DHS, Dortmund Health Study; KORA, Cooperative Health Research in the Region of Augsburg; NR, not reported; OR, odds ratio; PR, prevalence ratio; SHIP, Study of Health in Pomerania; WHS, Women’s Health Study.

### Risk of having migraine in obese subjects

**Figure 1:**
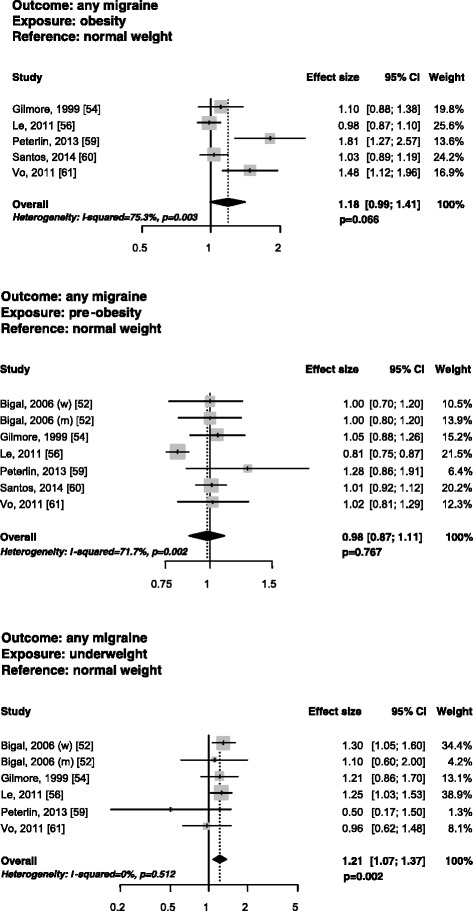
**Forest plots of the risk of having any migraine in obese, pre-obese, or underweight vs. normal weight subjects in studies fulfilling narrow inclusion criteria.**

**Figure 2:**
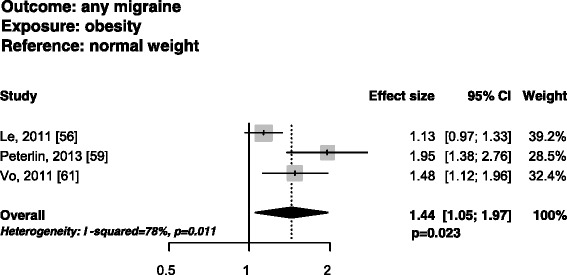
**Funnel plots of the risk of having any migraine in obese vs. normal weight women in studies fulfilling narrow inclusion criteria.**

Five cross-sectional studies fulfilling the narrow inclusion criteria [54,56,59-61] investigated the risk of having migraine in obese subjects as compared with normal weight subjects. Two studies [59,61] found a significant association between the two variables, while the other three [54,56,60] did not find any association. Regarding women, two studies [59,61] found an increased risk of having any migraine in obese as compared with normal weight women while another study [56] did not find any association. In men, no increase was found in the risk of having any migraine in obese subjects as compared with normal weight subjects [56,59]. The pooled analysis did not suggest an increased risk of having migraine in obese as compared with normal weight subjects overall (PAEE 1.18; 95% CI, 0.99-1.41; P = 0.066), with substantial statistical heterogeneity (I^2^ = 75.3%; P = 0.003) (Figure 1). In obese women, the risk of having migraine was increased (PAEE 1.44; 95% CI, 1.05-1.97; P = 0.023), with substantial statistical heterogeneity (I^2^ = 78.0%; P = 0.011) (Figure 2), while the same risk was not increased in obese men (PAEE 1.04; 95% CI, 0.86-1.25; P = 0.715), with no statistical heterogeneity (I^2^ = 0%; P = 0.350) (Additional file 2).

Two additional cross-sectional studies fulfilling narrow inclusion criteria [57,58] defined obesity as a dichotomous variable with a BMI cut-off of 30 kg/m^2^. One of the studies [58] found an increased risk of having migraine only in obese subjects aged 55 years or less and no increased risk in obese subjects aged over 55 years, as compared with non-obese subjects. The other study [57], which was conducted in women aged 40 to 74 years, did not find any risk. The pooled analysis did not suggest an increased risk of having migraine in obese as compared with non-obese subjects (PAEE 1.11; 95% CI, 0.89-1.39; P = 0.365), with substantial statistical heterogeneity (I^2^ = 81.7%; P = 0.0002) (Additional file 2).

Four additional studies [48,49,51,52] met broad inclusion criteria; among them, only one [51] found an increased risk of having migraine in obese compared with normal weight subjects. The pooled analysis including those studies suggested an increased risk of having migraine in obese compared with normal weight subjects (PAEE 1.14; 95% CI, 1.02-1.27; P = 0.017), with moderate statistical heterogeneity (I^2^ = 63.3%; P for Q test = 0.002).

### Risk of having migraine in pre-obese subjects

Six cross-sectional studies fulfilling narrow inclusion criteria, in seven cohorts [52,54,56,59-61], investigated the risk of having migraine in pre-obese as compared with normal weight subjects. One study [56] found a decreased risk of having migraine in pre-obese subjects while the other studies [52,54,59-61] did not find any increase in the risk. In women, one study [59] found an increased risk of having migraine in pre-obese as compared with normal weight women, while three studies [52,56,61] did not find an increase in the same risk. In men, one study [56] found a decrease in the same risk while two other studies [52,58] did not find any risk. The pooled analysis did not suggest an increased risk of having migraine in pre-obese as compared with normal weight subjects (PAEE 0.98; 95% CI, 0.87-1.11; P = 0.767), with substantial statistical heterogeneity (I^2^ = 71.7%; P = 0.002) (Figure 1). In pre-obese women, the risk of having migraine was not increased (PAEE 1.08; 95% CI, 0.93-1.24; P = 0.308), with moderate statistical heterogeneity (I^2^ = 42.4%; P = 0.157), while in pre-obese men the risk was decreased (PAEE 0.90; 95% CI, 0.81-1.00; P = 0.040), without statistical heterogeneity (I^2^ = 0%; P = 0.409) (Additional file 2).

Three additional studies [48,50,51] met broad inclusion criteria; none of them found an increased risk of having migraine in pre-obese compared with normal weight subjects. The pooled analysis including those studies confirmed the non-increased risk of having migraine in pre-obese compared with normal weight subjects (PAEE 0.99; 95% CI, 0.89-1.10; P = 0.870), with moderate statistical heterogeneity (I^2^ = 66.0%; P for Q test = 0.001).

### Risk of having migraine in underweight subjects

Five cross-sectional studies fulfilling narrow inclusion criteria, in six cohorts [52,54,56,59,61], investigated the risk of having migraine in underweight as compared with normal weight subjects. One [56] of the four studies [54,56,59,61] reporting the result for the overall population found a significant association between the two variables, while the other three studies [54,59,61] did not find any association. Four studies reported the results by gender [52,56,59,61]. One of them [52] found an increased risk of having any migraine in underweight as compared with normal weight women, while the others [56,59,61] did not find any increased risk. In men, no increase was found in the risk of having any migraine in underweight as compared with normal weight men [52,56]. The pooled analysis suggested an increased risk of having migraine in underweight as compared with normal weight subjects (PAEE 1.21; 95% CI, 1.07-1.37; P = 0.002), without statistical heterogeneity (I^2^ = 0%; P = 0.512) (Figure 1). The risk of having migraine in underweight women (PAEE 1.15; 95% CI, 0.97-1.36; P = 0.101) or men (PAEE 1.15; 95% CI, 0.75-1.76; P = 0.529) was not increased, with low (I^2^ = 17.7%; P = 0.303) and absent (I^2^ = 0%; P = 0.843) statistical heterogeneity, respectively (Additional file 2).

One additional study [51] met broad inclusion criteria; that study did not find an increased risk of having migraine in underweight compared with normal weight subjects. The pooled analysis including that study confirmed the increased risk of having migraine in underweight compared with normal weight subjects (PAEE 1.21; 95% CI, 1.08-1.37; P = 0.017), with moderate statistical heterogeneity (I^2^ = 63.3%; P for Q test = 0.002).

### Risk of having chronic migraine according to BMI status

**Figure 3:**
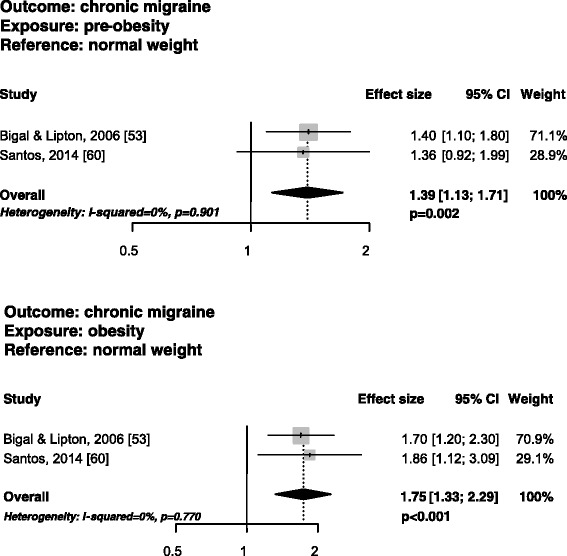
**Funnel plots of the risk of having chronic migraine in pre-obese vs. normal weight subjects in studies fulfilling narrow inclusion criteria.**

Two cross-sectional studies fulfilling narrow inclusion criteria [53,60] investigated the risk of having chronic migraine in pre-obese as compared with normal weight subjects. One of the two studies [53] found an increased risk of having chronic migraine in pre-obese subjects, while the other [60] did not find any risk. The pooled analysis suggested an increased risk of having chronic migraine in pre-obese as compared with normal weight subjects (PAEE 1.39; 95% CI, 1.13-1.71; P = 0.002), without statistical heterogeneity (I^2^ = 0%; P = 0.901) (Figure 3). Those same two studies [53,60] investigated the risk of having chronic migraine in obese as compared with normal weight subjects; however, one of the two studies [53] only fulfilled broad inclusion criteria. The pooled analysis suggested an increased risk of having chronic migraine in obese compared with normal weight subjects (PAEE 1.75; 95% CI, 1.33-2.29; P < 0.001), without statistical heterogeneity (I^2^ = 0%; P for Q test = 0.770) (Figure 3).

### Risk of being obese in migraineurs

One prospective cohort [62] and one cross-sectional study [55] fulfilling narrow inclusion criteria investigated the risk of being obese in migraineurs as compared with non-migraineurs. The former [62] did not find any association between migraine and obesity while the latter [55] found an increased risk of being obese in migraineurs.

The pooled analysis did not suggest an increased risk of being obese in migraineurs as compared with non-migraineurs (PAEE 1.28; 95% CI, 0.74-2.21; P = 0.374), with substantial statistical heterogeneity (I^2^ = 82.8%; P = 0.016) (Additional file 2).

### Linear analysis

Linear analysis performed using the generalized least-squares method on the studies fulfilling broad inclusion criteria showed that the effect size associated with every 5 kg/m^2^ increase in BMI was 0.993 (95% CI, 0.934-1.056, P = 0.818), with substantial statistical heterogeneity (I^2^ = 66.6%; P < 0.001).

### Sensitivity analyses

The “leave-one-out” analysis revealed no substantial changes after the exclusion of each study from the pooled analyses in terms of the risk of having migraine in underweight or pre-obese subjects as compared with normal weight subjects, while the risk of migraine in obese as compared with normal weight subjects became significant after the exclusion of one study [56] from the pooled analysis (data not shown).

None of the cross-sectional studies fulfilling narrow inclusion criteria was of low quality according to the NOS; therefore, we did not perform sensitivity analyses based on study quality. On the contrary, the included cohort study [62] did not fulfill three of the NOS criteria (see Additional file 3), indicating a high risk of bias; however, we could not perform sensitivity analyses with the exclusion of that study because it was included in a pooled analysis with only one other study.

## Discussion

When including observational studies reporting the standard WHO BMI categories, our meta-analysis suggested that the risk of having migraine in underweight subjects was increased by about one fifth as compared with normal weight subjects; the risk was increased also in obese as compared with normal weight women, while it was decreased in pre-obese as compared with normal weight men. When including studies with different BMI cut-offs (broad inclusion criteria), we additionally found a slightly increased risk of having migraine in obese as compared with normal weight subjects. Notably, one of the additional studies was performed in a female population [49], while the other [48,50,51] investigated populations with more women than men; therefore, in the pooled analysis a specific effect of obesity on female migraineurs may have been more evident. Together with age and attack frequency, gender is one of the factors potentially modifying the association between migraine and body weight; regrettably, only a minority of the available studies adequately consider those factors, and with inconsistent results. Data about possible gender differences are also scarce in our meta-analysis, since several studies did not report separate analyses by gender [48,50,54,55,60]. Additionally, as migraine is more frequent in women than in men, studies performed in men may be underpowered to detect any significant association.

Age may be another important factor influencing the effect of obesity on migraine: studies performed in younger subjects [58,59,61] found an increased risk of migraine in obese subjects, while no association was found between obesity and migraine in older subjects [57,58]. The negative results reported on the association between migraine and obesity by most studies were attributed to the inclusion of subjects of different ages, and to the possible role of pre-menopausal hormonal status in contributing to the association [58,59,61]. Patterns of migraine activity, which is maximal in young adults and declines after the age of 40 [63], could be another factor influencing the association.

We found a markedly increased risk of chronic migraine in overweight subjects as compared with normal weight subjects. The finding of an increased risk of having chronic migraine in pre-obese as compared with normal weight subjects, though taken from the pooled analysis of only two studies [53,60], had no statistical heterogeneity. Moreover, the pooled analysis of those same studies, which had different BMI cut-offs for obesity, showed an even more remarkable increased risk of chronic migraine in obese compared with normal weight subjects. Some studies have also found an association between obesity and chronic daily headache [64] or frequent headaches [14], suggesting that increased body weight is a risk factor for chronification of non-migraine headaches; however, evidence suggests that this is not valid for chronification of tension-type headaches [6,52], while other data suggest that frequent intake of acute pain drugs may also have a role in migraine chronification [47]. Therefore, although an association between chronic migraine and body weight may exist, it is unclear whether that association is direct or mediated by other factors.

Some studies support an association between pre-obesity or obesity and increased attack frequency or chronic migraine [6,49,62]. Notably, among them, a large cross-sectional study found a higher migraine frequency in underweight, pre-obese, and obese as compared with normal weight subjects; the association was more evident, according to the authors, for daily migraine [49]. In a prospective follow-up of the same cohort [62], higher migraine frequency was not associated with the development of overweight or obesity suggesting that obesity may favor migraine chronification while the inverse relationship is not valid. However, other studies did not show any association between migraine frequency and obesity [29,59,61].

While several studies are available to examine the risk of having migraine in obese or pre-obese subjects, fewer studies [45,46,55,62] have assessed the risk of being pre-obese or obese in migraineurs as compared with non-migraineurs. Two such studies [45,46] were excluded from the analyses; one of those two studies [45] did not find an increased risk of obesity in migraineurs as compared with non-migraineurs, while the other [46] found an increased risk of migraine with and without aura in obese as compared with non-overweight subjects.

As recommended by the Cochrane Collaboration, the eligibility criteria for a meta-analysis should be sufficiently broad to encompass the likely diversity of studies, but sufficiently narrow to ensure that a meaningful answer can be obtained when studies are considered in aggregate [15]. We performed our primary analyses only on studies with uniform BMI cut-offs (narrow criteria) in order to reduce between-studies heterogeneity; however, those analyses may have excluded too many studies; therefore, we performed additional analyses with broad inclusion criteria.

The studies excluded from our analyses because they included headaches other than migraine as an outcome [14,19-22] found either a linear association between BMI and the risk of having headache [14,21] or an association between underweight [19] or obesity [19,22], but not pre-obesity, and the risk of having headache. However, in one study [20] the risk of having headache in underweight or obese compared with normal weight subjects became non-significant in multivariate analyses. Taken together, those findings suggest that non-migrainous headaches are more influenced by body weight than migraine. Several considerations led us to exclude headache disorders other than migraine from the analyses. Firstly, some headache disorders, such as idiopathic intracranial hypertension, have been shown to be closely associated with obesity [65]; therefore, a positive association between headache and obesity would likely have been driven by those specific disorders. Secondly, previous studies have shown that migraine and tension-type headache have different patterns of association with body weight [53]. Thirdly, migraine and obesity are both associated with increased risk of vascular diseases [66], which adds interest to the investigation of their association.

The worsening of migraine induced by obesity may have an inflammatory origin. Adipocytes release adipocytokines, and particularly adiponectin, which induces a pro-inflammatory state and may activate the nitric oxide pathway in the brain, thus causing or worsening headache disorders [67]. Markers of oxidative stress and metabolic risk such as nitrates and oxidized low-density lipoproteins have been found to be increased in migraineurs as compared with non-migraineurs [68] and may represent a molecular link between migraine and obesity [69]. Several studies have assessed the levels of adipocytokines in subjects with migraine, with conflicting results [70]. The adipose tissue releases proinflammatory cytokines such as tumor necrosis factor alpha (TNFα), interleukin (IL)-1, and IL-6, whose levels were also increased in migraineurs [71]. Chronic inflammatory states have been found in the metabolic syndrome, of which obesity is part [72], and an increased risk of metabolic syndrome was found in migraineurs as compared with non-migraineurs [37], potentially explaining the associations between migraine, obesity, and overall vascular risk.

In the association between migraine and underweight, psychiatric comorbidities such as anxiety and depression could act as potential confounders [73]; psychiatric comorbidities could also influence the relationship between migraine and obesity [74]. Moreover, in the subgroup analyses by gender we did not find any association between migraine and underweight either in men or in women, suggesting the possibility of a spurious interaction in the overall population.

To the best of our knowledge, this is the first quantitative assessment of the associations between obesity, pre-obesity or underweight, and migraine. The studies included in the analyses were of good average quality and investigated large populations.

Nonetheless, several issues should be raised. The exclusion of non-English studies may have introduced bias, although when reviewing titles and abstracts of retrieved studies we did not find any study in a non-English language fulfilling all the other inclusion criteria. In addition, despite the numerous available data only a few studies considered homogeneous BMI categories that could be included in the pooled analyses. The included studies had important sources of clinical heterogeneity (differences in age range and gender distribution of the included subjects) and methodological heterogeneity (confounders, definitions of migraine). The identification of migraine subtypes was reported in only three of the retrieved studies [46,56,62] and these had different designs (cross-sectional and cohort) and BMI category definitions, so could not be included in a pooled analysis. In most cases migraine was self-reported, thus potentially introducing a recall bias. The available studies did not consider the effect on weight gain of preventive migraine agents usually prescribed in case of high headache frequency [75]. The utilization of these drugs may partly explain the association between high headache frequency and obesity. Finally, BMI does not take into account the difference between lean and fat body mass. Therefore, care must be taken not to classify a healthy, muscular individual with very low body fat as overweight or obese using the BMI formula. Other parameters, such as abdominal obesity, may be more informative on the real metabolic status of a subject, and may have a different distribution pattern in the general population than BMI [76].

## Conclusions

Our meta-analysis of observational studies suggests an association between migraine and obesity likely conditioned by female gender. Evidence also suggests that pre-obesity or obesity are risk factors for frequent or chronic migraine. Further research should take into account potential modifiers of the association between migraine and body weight such as gender, age, migraine characteristics (duration, frequency, activity, severity, and disability), together with migraine diagnostic criteria and potential confounders such as medication intake and migraine comorbidities. The association between migraine and body weight has potential clinical implications; as suggested by the available data, body weight management could be relevant for prevention of migraine chronification and for improving the availability of alternative treatment options.

## Additional files

Additional file 1:
**Flowchart of study selection.**
Additional file 2:
**Funnel plots of further analyses.**
Additional file 3:
**Quality analysis of studies fulfilling narrow inclusion criteria.**
