# Supplementary material for: Migraine and body mass index categories: a systematic review and meta-analysis of observational studies
Source: J Headache Pain. 2015 Mar 28;16:27. doi: 10.1186/s10194-015-0510-z (PMC4385329; doi:10.1186/s10194-015-0510-z)
Supplement: Additional file 1: — Flowchart of study selection. [file 10194_2015_510_MOESM1_ESM.doc]

Total records found: 2,022

PubMed: 929
Science Citation Index: 881

Scopus: 212

520 duplicates

1,502 Title/Abstract review

1,461 Excluded studies

- 1,317 did not pertain to the addressed topic
- 144 papers with other designs (e.g. review, letter, case series)

5 added by hand search

46 Full-text review

31 Excluded studies

- 6 did not define or assess the variables of interest
- 21 did not report extractable data on the variables of interest
- 1 performed only in adolescents
- 1 not defining obesity by means of BMI categories
- 2 not comparable with others

15 Included studies

11 Narrow criteria (obesity defined according to WHO BMI categories for Western people)

- 1 Cohort
- 10 Cross-sectional

~~4~~ Broad criteria (obesity not defined according to WHO BMI categories for Western people)

- 3 Cross-sectional (non-WHO BMI categories)
- 1 Cross-sectional (WHO BMI categories for Asian people)
