# Supplementary material for: Migraine and body mass index categories: a systematic review and meta-analysis of observational studies
Source: J Headache Pain. 2015 Mar 28;16:27. doi: 10.1186/s10194-015-0510-z (PMC4385329; doi:10.1186/s10194-015-0510-z)
Supplement: Additional file 2: — Funnel plots of further analyses. [file 10194_2015_510_MOESM2_ESM.pdf]

Outcome: any migraine
Exposure: obesity
Reference: non-obese

Study	Effect size	95% CI	Weight
Mattsson, 2007 [57]	1.10	[0.60; 1.80]	10.4%
Peterlin, 2010 (m≤55) [58]	1.38	[1.20; 1.59]	25.4%
Peterlin, 2010 (m>55) [58]	0.77	[0.54; 1.10]	16.5%
Peterlin, 2010 (w≤55) [58]	1.39	[1.24; 1.55]	26.4%
Peterlin, 2010 (w>55) [58]	0.86	[0.68; 1.10]	21.3%
**Overall**	**1.11**	**[0.89; 1.39]**	**100%**
Heterogeneity: I-squared=81.7%, p=0.0002	**p=0.365**		

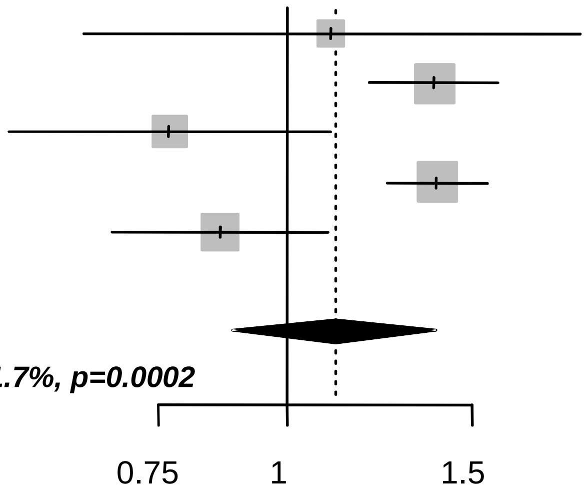

Women

Outcome: any migraine
Exposure: pre-obesity
Reference: normal weight

Study	Effect size	95% CI	Weight
Bigal, 2006 [52]	1.00	[0.70; 1.20]	19.1%
Le, 2011 [56]	1.03	[0.93; 1.14]	45.5%
Peterlin, 2013 [59]	1.58	[1.10; 2.27]	12.4%
Vo, 2011 [61]	1.02	[0.81; 1.29]	23.1%
**Overall**	**1.08**	**[0.93; 1.24]**	**100%**

Heterogeneity: I - squared=42.4%, p=0.157

p=0.308

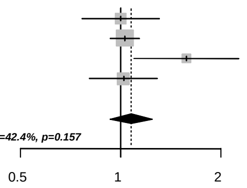

Outcome: any migraine
Exposure: underweight
Reference: normal weight

Study	Effect size	95% CI	Weight
Bigal, 2006 [52]	1.30	[1.05; 1.60]	43.0%
Le, 2011 [56]	1.12	[0.90; 1.39]	41.2%
Peterlin, 2013 [59]	0.56	[0.19; 1.70]	2.3%
Vo, 2011 [61]	0.96	[0.62; 1.48]	13.4%
**Overall**	**1.15**	**[0.97; 1.36]**	**100%**

Heterogeneity: I -squared=17.7%, p=0.303

p=0.101

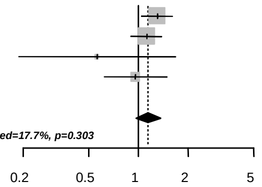

Men

Outcome: any migraine
Exposure: obesity
Reference: normal weight

Study	Effect size	95% CI	Weight
Le, 2011 [56]	1.01	[0.83; 1.23]	92.7%
Peterlin, 2013 [59]	1.43	[0.71; 2.89]	7.3%
**Overall**	**1.04**	**[0.86; 1.25]**	**100%**

Heterogeneity: I - squared=0%, p=0.350

p=0.715

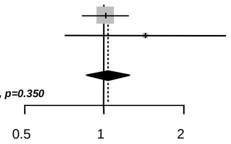

Outcome: any migraine
Exposure: pre-obesity
Reference: normal weight

Study	Effect size	95% CI	Weight
Bigal, 2006 [52]	1.00	[0.80; 1.20]	25.7%
Le, 2011 [56]	0.87	[0.77; 0.98]	72.6%
Peterlin, 2013 [59]	0.69	[0.32; 1.51]	1.8%
**Overall**	**0.90**	**[0.81; 1.00]**	**100%**

Heterogeneity: I - squared=0%, p=0.409

p=0.040

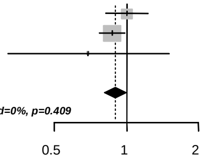

Outcome: any migraine
Exposure: underweight
Reference: normal weight

Study	Effect size	95% CI	Weight
Bigal, 2006 [52]	1.10	[0.60; 2.00]	51%
Le, 2011 [56]	1.20	[0.65; 2.22]	49%
**Overall**	**1.15**	**[0.75; 1.76]**	**100%**

Heterogeneity: I -squared=0%, p=0.843

p=0.529

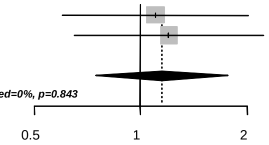

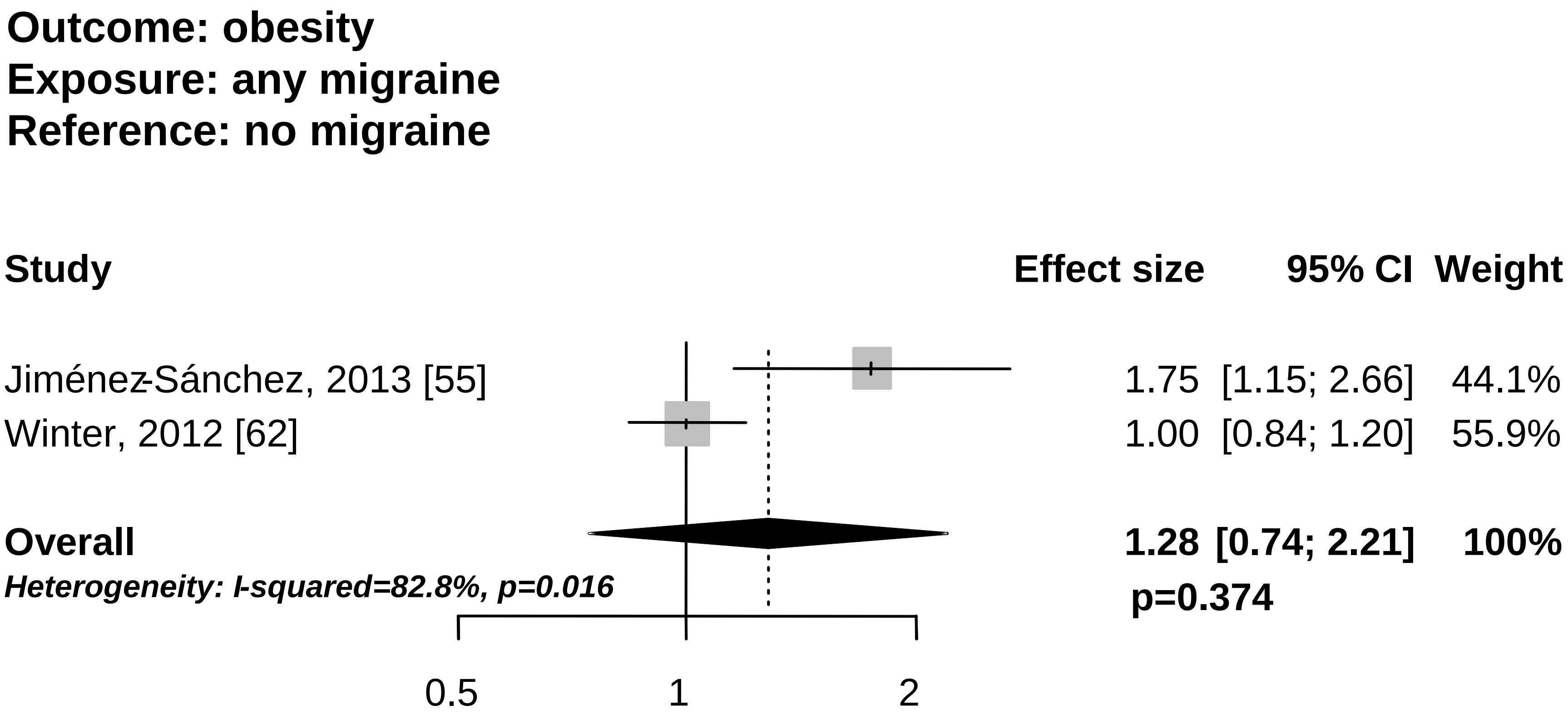
Outcome: obesity
Exposure: any migraine
Reference: no migraine
Study
Effect size
95% CI
Weight
Jiménez-Sánchez, 2013 [55]
1.75 [1.15; 2.66] 44.1%
Winter, 2012 [62]
1.00 [0.84; 1.20] 55.9%
Overall
1.28 [0.74; 2.21] 100%
Heterogeneity: I-squared=82.8%, p=0.016
p=0.374
0.5
1
2
