# Supplementary material for: Migraine and body mass index categories: a systematic review and meta-analysis of observational studies
Source: J Headache Pain. 2015 Mar 28;16:27. doi: 10.1186/s10194-015-0510-z (PMC4385329; doi:10.1186/s10194-015-0510-z)
Supplement: Additional file 3: — Quality analysis of studies fulfilling narrow inclusion criteria. [file 10194_2015_510_MOESM3_ESM.doc]

**Online Table. Assessment of risk of bias within the cross-sectional (a) and cohort (b) studies fulfilling narrow inclusion criteria**

**a)**

| **First author, year** | **Representativeness of the sample** | **Sample size** | **Non-respondents** | **Ascertainment of the exposure** | **Comparability** | **Assessment of the outcome** | **Statistical test** |
| --- | --- | --- | --- | --- | --- | --- | --- |
| Bigal, 2006 [52] | * | * | - | * | ** | * | * |
| Bigal and Lipton, 2006 [53] | * | * | - | * | ** | * | * |
| Gilmore, 1999 [54] | * | * | - | * | ** | * | * |
| Jiménez-Sánchez, 2013 [55] | * | - | - | * | * | * | * |
| Le, 2011 [56] | - | * | - | * | ** | * | * |
| Mattsson, 2007 [57] | - | * | - | * | ** | * | * |
| Peterlin, 2010 [58] | * | * | - | ** | ** | * | * |
| Peterlin, 2013 [59] | * | * | - | * | ** | * | * |
| Santos, 2014 [60] | * | * | - | ** | ** | * | * |
| Vo, 2011[61] | - | * | - | * | ** | * | * |

** quality criterion completely satisfied; * quality criterion satisfied; - quality criterion not satisfied or insufficient information to adjudicate as satisfied

**b)**

| **First author, year** | **Representativeness of the exposed cohort** | **Selection of the non-exposed cohort** | **Ascertainment of exposure** | **Outcome not present at start** | **Comparability of cohorts** | **Assessment of outcome** | **Follow-up long enough for outcomes to occur** | **Adequate follow-up of cohorts** |
| --- | --- | --- | --- | --- | --- | --- | --- | --- |
| Winter, 2012 [62] | - | * | - | * | ** | - | * | * |

** quality criterion completely satisfied; * quality criterion satisfied; - quality criterion not satisfied or insufficient information to adjudicate as satisfied
